# Vulnerability of the developing brain to hypoxic-ischemic damage: contribution of the cerebral vasculature to injury and repair?

**DOI:** 10.3389/fphys.2012.00424

**Published:** 2012-11-09

**Authors:** Ana A. Baburamani, C. Joakim Ek, David W. Walker, Margie Castillo-Melendez

**Affiliations:** ^1^The Ritchie Centre, Monash Medical Centre, Monash Institute of Medical Research, ClaytonMelbourne, VIC, Australia; ^2^Sahlgrenska Academy, Gothenburg UniversityGöteborg, Sweden; ^3^Department of Obstetrics and Gynaecology, Monash University, ClaytonMelbourne, VIC, Australia

**Keywords:** hypoxia, cerebral blood vessels, angiogenesis, hemorrhage, blood-brain barrier

## Abstract

As clinicians attempt to understand the underlying reasons for the vulnerability of different regions of the developing brain to injury, it is apparent that little is known as to how hypoxia-ischemia may affect the cerebrovasculature in the developing infant. Most of the research investigating the pathogenesis of perinatal brain injury following hypoxia-ischemia has focused on excitotoxicity, oxidative stress and an inflammatory response, with the response of the developing cerebrovasculature receiving less attention. This is surprising as the presentation of devastating and permanent injury such as germinal matrix-intraventricular haemorrhage (GM-IVH) and perinatal stroke are of vascular origin, and the origin of periventricular leukomalacia (PVL) may also arise from poor perfusion of the white matter. This highlights that cerebrovasculature injury following hypoxia could primarily be responsible for the injury seen in the brain of many infants diagnosed with hypoxic-ischemic encephalopathy (HIE). Interestingly the highly dynamic nature of the cerebral blood vessels in the fetus, and the fluctuations of cerebral blood flow and metabolic demand that occur following hypoxia suggest that the response of blood vessels could explain both regional protection and vulnerability in the developing brain. However, research into how blood vessels respond following hypoxia-ischemia have mostly been conducted in adult models of ischemia or stroke, further highlighting the need to investigate how the developing cerebrovasculature responds and the possible contribution to perinatal brain injury following hypoxia. This review discusses the current concepts on the pathogenesis of perinatal brain injury, the development of the fetal cerebrovasculature and the blood brain barrier (BBB), and key mediators involved with the response of cerebral blood vessels to hypoxia.

## Introduction

The most devastating permanent injury that can occur in the developing fetus is germinal matrix-intraventricular hemorrhage (GM-IVH) with the incidence greater in premature and low birth weight babies. Term infants are more susceptible to breakdown of the blood brain barrier (BBB), brain edema, subcortical hemorrhage and impaired metabolism (Wigglesworth and Pape, 1978; Van den Broeck et al., 2007). Volpe (1989) suggests that consequences and subsequent neuropathology that originate from GM-IVH include destruction of the germinal matrix, periventricular hemorrhagic infarction (possibly of venous origin), periventricular leukomalacia (PVL), hydrocephalus and pontine neuronal necrosis. Analysis of neonates with PVL suggest that this area of infarct contained poorly perfused vessels of an irregular capillary shape, suggesting a relationship between vascular supply to this region and the pathology of PVL (Takashima and Tanaka, 1978a).

Clinicians have long sought to understand the inherent vulnerability of the sub-ependymal germinal matrix to hemorrhage and periventricular white matter to necrosis. Observations of infarctions in these periventricular areas were seen to occur in watershed vascular or border “end zones” (areas where long and penetrating arteries end). These areas were deemed more vulnerable to poor perfusion and less capable of tolerating fluctuations of cerebral blood flow and cerebral venous pressure especially following hypoxia-ischemia and/or reperfusion (De Reuck, 1971; Takashima and Tanaka, 1978a,b; Wigglesworth and Pape, 1978; Volpe, 1989).

Ballabh (2010) suggests that the germinal matrix, which is highly vascularized, may have an inherent fragility during development deeming it vulnerable to hemorrhage, which may be further exacerbated following hypoxia. Interestingly, Volpe (1989) also highlights that many thin walled cerebral blood vessels are the source of bleeding. This finding was also seen in the beagle puppy model of germinal matrix hemorrhage (Trommer et al., 1987; Ment et al., 1991) but not in the rhesus monkey (Lenn and Whitmore, 1985). The observation of thin walled blood vessels could be indicative of structural immaturity or ongoing angiogenesis. However, Back et al. consider that injury occurring in periventricular white matter is more likely to be due to cellular immaturity—especially of oligodendrocyte progenitors—than of the vascular elements within these regions (Back et al., 2007; McClure et al., 2008).

The response of the cerebrovasculature following hypoxia-ischemia and how this may contribute to perinatal brain injury is not well investigated. In the adult brain, robust up-regulation of vascular endothelial growth factor (VEGF) occurs following cerebral hypoxia and/or ischemia which, as an adaptive response, results in the formation of new blood vessels. However, newly-formed blood vessels are fragile and prone to rupture. The observation of thin walled blood vessels in regions prone to bleeding and hemorrhage (Volpe, 1989), could be indicative of structural immaturity or ongoing angiogenesis. Incomplete development of hypoxia-induced angiogenesis could be a major factor in the cerebral injury that follows the birth of a severely hypoxic fetus, or which arises in the neonate where resuscitation is difficult and prolonged. The response of blood vessels, changes in blood flow, and the reactive expression of proteins by the vasculature could be directly affected by the processes of excitotoxicity and oxidative stress which are already known to be closely inter-related, and the occurrence of hemorrhage and leukomalacia highlight that the response of the vasculature is pivotal. Understanding the development and response of the cerebrovasculature and BBB could reveal reasons for regional vulnerability of the immature brain to vascular damage.

## Perinatal hypoxia

Intrapartum asphyxia is present in up to 25 per 1000 live births in term deliveries (Low, 2004). However, the incidence of antepartum and/or intrauterine asphyxia is much more difficult to determine. Post mortem studies indicate that hypoxic-ischemic and asphyxic episodes that occur during pregnancy significantly contribute to brain injury, morbidity and mortality (Sims et al., 1985; Low et al., 1989; Low, 2004). Intrauterine asphyxia also contributes to cognitive impairment, developmental delay, epilepsy, motor deficits and cerebral palsy (Low et al., 1989; Cowan et al., 2003; Low, 2004; Glass et al., 2009). The Australian Cerebral Palsy Foundation states that the incidence of cerebral palsy (approximately 1 in 400 live births) has remained relatively unchanged over the last 40 years, despite reduction in the rate of perinatal morbidity (Stanley and Watson, 1992). This highlights the importance of understanding the contribution of intrauterine events, such as fetal hypoxia, academia, and asphyxia to the pathogenesis of brain injury.

Asphyxia describes an insufficiency to exchange respiratory gases (Stephens, 1995; Parer, 1998). The severity of asphyxia and hypoxia-ischemia are thereby defined as a series of cardio-respiratory changes, including hypoxia (decreased pO2), hypercapnia (increased pCO2), hypoxemia, metabolic acidosis (increased lactate), impaired blood gas exchange and ischemia decreased oxygen delivery to tissue. Some argument exists over whether “asphyxia” can be applied to events occurring during gestation since, in the adult, asphyxia describes deficient oxygen supply arising from the inability to breathe (such as seen during strangulation). In utero the fetus does not breathe, but fetal asphyxia does arise from insufficient umbilical or uterine blood flow resulting in impaired blood gas exchange (Parer, 1998; Low, 2004), and is therefore, used to describe the simultaneous decrease in oxygen and increase in carbon dioxide levels in fetal blood and tissues. However, without measurement of blood gases and cardiovascular parameters, the definition of asphyxia is difficult, and in this review of fetal and neonatal pathophysiology the terms hypoxia or hypoxia-ischemia will be used. Common causes of hypercapnic hypoxia in the fetus include maternal infection, hypertension, diabetes and placental insufficiency, all of which have the tendency to reduce uterine and/or umbilical blood flows. Fetal factors can range from multiple births, tangling or obstruction of the umbilical cord, or partial separation of the placenta from the uterine wall. The extent of injury that can occur as a consequence of hypoxia are dependent on the duration, degree and frequency of the insult. The gestational age at which hypoxia occurs also affects the brain regions involved.

### Perinatal brain injury

Hypoxia is one of the most common causes of neonatal brain injury, and it still remains to be well understood as to why some brain regions are more vulnerable to injury than others. The brain regions susceptible to hypoxic injury also change as the infant matures (Barkovich, 2005). A possible explanation for the correlation between gestational age and the type of brain injury may be the changing location of intervascular “watershed” boundary zones. From human infant autopsy tissue it was found the most prominent injury that occurred was hemorrhage with areas of necrotic tissue, and with exacerbated injury in neonates that had suffered hypoxia-ischemia or a complicated pregnancy. A recent study demonstrated that hypoxic-ischemic injury in term infants resulted in subcortical hemorrhage in 13% of the subjects, while 24% of preterm infants showed IVH (Van den Broeck et al., 2007). From studies in fetal sheep it has been shown that younger fetuses can tolerate longer periods of complete cessation of umbilical blood flow, up to 25 min, however, near-term fetuses can only withstand up to 10 min (Mallard et al., 1994; Keunen et al., 1997). The differing tolerance across gestation to duration is suggested to be due to the greater tissue metabolic rate of the near term fetus, and hence the greater response in terms of excitotoxicity, free radical production, and propensity of mitochondrial damage (Bennet et al., 1999). To understand the impact hypoxia has on the developing brain, the most prominent types of brain injury are described below.

#### Preterm brain injury

Premature neonates who are also of a low birth weight are at the highest risk of PVL and GM-IVH. The source of GM-IVH was classified by Takashima and Tanaka (1978b), Wigglesworth and Pape (1978), and Volpe (1989) who described bleeding occurring around the subependymal germinal matrix (around the lateral ventricle) extending into the white matter. PVL is characterized by hemorrhagic necrosis of the white matter surrounding the lateral ventricles. It can be diffuse or focal and commonly occurs in the preterm infant [reviewed by Volpe (1998)]. Cellular immaturity of oligodendrocytes also significantly contributes to regional vulnerability of white matter (Alvarez-Diaz et al., 2007; Back et al., 2007). This “selective vulnerability” governs the subsequent manifestation of injury. Blood vessels within the germinal matrix are fragile, particularly during the period of high cell turnover (24–32 weeks gestation) associated with corticogenesis. At this point of gestation, the oxygen and nutrient demand of the germinal matrix is high, and the requirement for correspondingly high blood flow appears to be met by a network of very fragile and leaky blood vessels. GM-IVH is a common brain injury in premature infants, particularly at <30 weeks gestation, and is more common when babies suffer additional stresses such as respiratory distress syndrome, pneumothorax, or high blood pressure, although it can also occur in healthy premature and full-term babies. IVH implies structural immaturity of blood vessels in the germinal matrix, and is associated with deficient autoregulatory capacity and pressure-passive cerebral perfusion (Pryds et al., 1989). Elevation of intracerebral venous pressure may be involved, as obstructed venous drainage has been shown to be associated with periventricular hemorrhage (Perlman, 1998).

The developmental status of the cerebral vasculature is also thought to be a critical predisposing factor in the pathogenesis on PVL. The topographic predilection of white matter injury is distinct between immature and mature brains, because regional cerebral blood flow and metabolic demand are rapidly changing during the perinatal period. In the immature brain, the end-zones of perforating arteries through the cerebral cortex are located in the periventricular white matter, which is a watershed area vulnerable to PVL. A predisposing factor in the pathogenesis of IVH in preterm infants is the microvascular architecture of the germinal matrix, which is at the end zone of thalamo-striatal arteries and in the venule structure zone of the ventricular side of terminal vein (Takashima et al., 1978). Hypotension or hypoxemia may induce focal hypoxic-ischemic changes in the arterial end zone within the germinal matrix, and with reperfusion or over-perfusion following hypoxia-ischemia, venous hemorrhage may occur due to the paucity of supportive connective tissue (Kamei et al., 1992). In addition, the microvasculature of the germinal matrix is frail because of an abundance of angiogenic blood vessels (Ballabh et al., 2007) that possess few pericytes, an incomplete and immature basal lamina, and an investment of astrocytic processes (“end-feet”) with a deficiency of glial fibrillary acidic protein (GFAP) (El-Khoury et al., 2006). Pericyte coverage is less in the germinal matrix vascular bed than in cortical gray or white matter in human fetuses, premature infants (20–28 weeks gestation) and premature rabbit pups (Braun et al., 2007), consistent with their propensity for structural collapse and hemorrhage.

#### Term brain injury

Term neonates who undergo birth asphyxia or hypoxia-ischemia late in gestation tend to have injury in the deep gray matter, hippocampus, brainstem, and thalamic regions (Swarte et al., 2009). The most common injuries are parasagittal cerebral injury, and injuries to the basal ganglia, including thalamic bleeding with associated IVH (Van den Broeck et al., 2007). In recent years mild white matter injury has also been reported to occur in the term neonate, occurring near birth (Li et al., 2009; Swarte et al., 2009). Cowan et al. (2003) studied MRI and post mortem tissue of full term infants and found that the period immediately preceding birth was an important time when brain injury could arise. The vascular boundary zones in term infants lies between the anterior and middle cerebral arteries and between the middle and the posterior cerebral arteries. PVL and hemorrhage tend to occur in term neonates at a lower but still significant incidence (Wigglesworth and Pape, 1978; Inder and Volpe, 2000; Perlman, 2004; Takenouchi et al., 2012). Watershed injury can also occur with or without deep cortical involvement (Swarte et al., 2009).

#### Perinatal stroke

Perinatal stroke is defined as a cerebrovascular event that can occur between 28 weeks gestation and 28 days postnatal age. The incidence of perinatal stroke (thromboembolism) is approximately 1 in 4000 live births, with <5% also being associated with asphyxia (Estan and Hope, 1997; Lynch et al., 2002). Clinical studies from full term neonates that have experienced perinatal arterial stroke show a greater incidence of cerebral palsy, epilepsy, language delay, behavioral abnormalities, and brain injury to the basal ganglia, internal capsule Broca's and Wernicke's area. Increased stroke size also further increases the incidence of cerebral palsy (Lee et al., 2005).

Clinical manifestation of infants who have experienced stroke include seizure activity and apnea from the second postnatal day (Koelfen et al., 1995; Perlman, 2004), this can also persist to neonatal encephalopathy (Ramaswamy et al., 2004). Focal stroke in the term infant is most common with the presentation of seizure activity without associated encephalopathy. However, if neonatal encephalopathy also occurred, neurodevelopmental outcome significantly worsened (de Vries et al., 1997; Perlman, 2004; Ramaswamy et al., 2004).

### Pathophysiology of hypoxia-ischemia in the neonatal brain

The developing brains' response to global hypoxia-ischemia is a multi-step process. Within the first few hours, regionally-specific increases of cerebral blood flow occur, followed by decreases due to either regional vasoconstriction or the progressive collapse of cardiac output, with the subsequent onset of excitotoxicity, energy depletion and generation of free radicals collectively resulting in increased apoptotic and necrotic cell death, and edema formation (Wigglesworth and Pape, 1978; Bennet et al., 1998; Jensen et al., 1999; Shalak and Perlman, 2004; Ferrari et al., 2010a). A secondary phase of injury occurs in the following hours and days, resulting in a neuroinflammatory response, mitochondrial permeabilisation, reperfusion, and a loss of cerebral autoregulation which can also lead to increased free radical production (Inder and Volpe, 2000; Hamrick and Ferriero, 2003; McLean and Ferriero, 2004; Hagberg et al., 2009; Leonardo and Pennypacker, 2009). More recently it has been described that a tertiary phase of brain injury, occurring in the weeks to years following an insult, may also contribute to exacerbating injury and/or preventing repair when mechanisms such as an inflammatory response persist (Fleiss and Gressens, 2012).

#### CNS excitotoxicity

Excitotoxicity refers to cell death that occurs due to intracellular accumulation of calcium resulting from the prolonged opening of voltage-sensitive channels associated with excitatory amino acid receptors (Beal, 1992). Prolonged neuronal depolarization increases neurotransmitter release, but astrocytic re-uptake of glutamate becomes restricted due to low availability of ATP, so overstimulation of glutamate receptors occurs due to the presence of excess extracellular glutamate (McLean and Ferriero, 2004) leading, in some cases, to seizure activity (Jensen et al., 1998; Bennet et al., 2010). Glutamate and adenosine, whose receptors are expressed on excitatory neurons, are both implicated in excitatory mediated injury following hypoxia (McLean and Ferriero, 2004). In addition, neurons are vulnerable to hypoxia-induced injury due to their high dependence on oxygen. Neuronal glutamate release, re-uptake and resynthesis is a tightly regulated metabolic pathway that is closely coupled with cerebral glucose oxidation demand. Glutamate receptors are highly expressed during fetal and neonatal development, so any severe fluctuations, such as those resulting from hypoxia can have devastating consequences (Low, 2004; McLean and Ferriero, 2004).

#### Oxidative stress

Oxidative stress arises when reactive oxygen species (ROS) and/or reactive nitrative species (RNS) are generated at a rate that exceeds the capacity of the endogenous antioxidant systems to neutralize them. The developing brain is considered to be extremely vulnerable to free radical damage due to its high lipid content (O'Brien and Sampson, 1965), with the amount of polyunsaturated fatty acids increasing during gestation, relatively high oxygen consumption and therefore, a high capacity to generate ROS, and low concentrations and activity of the principal antioxidant enzymes (Mishra and Delivoria-Papadopoulos, 1999; McLean and Ferriero, 2004; Vannucci and Hagberg, 2004; Ikonomidou and Kaindl, 2011; Miller et al., 2012). During normal metabolism ROS are produced in low concentrations, and may act as signaling molecules to modulate vasodilatation and cerebral blood flow (Paravicini et al., 2004), but under conditions that result in tissue hypoxia the accumulation of ROS can trigger the mitochondrial release of pro-apoptotic proteins resulting in cell death (Morita-Fujimura et al., 2001). Whether ROS-mediated damage occurs specifically to vascular elements in the developing brain is unclear.

NO is a weak free radical, produced by the actions of nitric oxide synthase (NOS) isoforms, and has a number of physiological roles including the physiological modulation of cerebral blood flow, modulating cellular respiration and mitochondrial ROS production, as well as having antioxidant properties (Beltran et al., 2000; Li and Jackson, 2002). Three well known isoforms of NOS—neuronal NOS (nNOS), endothelial NOS (eNOS), and inducible NOS (iNOS). iNOS is principally produced in macrophages, microglia, endothelial cells, and astrocytes, nNOS in neurons and eNOS primarily in endothelial cells and following neonatal hypoxia-ischemia has been found in neurons (Ferriero et al., 1996; McLean and Ferriero, 2004; Kaur and Ling, 2009).

Following neonatal hypoxia-ischemia both nNOS and eNOS are up-regulated (Van den Tweel et al., 2005). Whilst nNOS knockout mice appear to be protected following neonatal hypoxia-ischemia (Ferriero et al., 1996), studies from eNOS knockout mice suggest eNOS may play a protective role hypoxia-ischemia in the adult brain (Huang et al., 1996), which may be due to eNOS influencing neural migration and outgrowth and acting as a downstream regulator of angiogenesis (Chen et al., 2005). The lipid peroxidation that occurs following acute hypoxia in the fetal brain (Ikeda et al., 1998; Castillo-Melendez et al., 2004) may be due to the generation of peroxynitrite (OONO), following the non-enzymatic combination of NO and superoxide (Tan et al., 1998). Lipid peroxidation affects immature oligodendrocytes in particular in the preterm fetal brain (Back et al., 1998), and may be a major factor in the white matter damage that can arise from hypoxic insult in the developing brain (Back et al., 1998; Baud et al., 2004).

#### Response of inflammatory cells

Cell death that is mediated by a neuroinflammatory response is complex. Resident microglia are some of the first cells to become “activated.” Amoeboid microglial cells, important for phagocytosis in the developing brain, are found in clusters in periventricular white matter and in close association with blood vessels in many brain regions (Fujimoto et al., 1989; Kaur et al., 2007). Microglia are the resident immune cells in the nervous system, whose presence is also related to vascularization, particularly in the early developmental stages of the human brain (Fujimoto et al., 1989). Microglia are also associated with active myelination during development (Hutchins et al., 1992; Ling and Wong, 1993; Kaur et al., 2007; Kaur and Ling, 2009). Activated microglia and astrocytes migrate to ischemically damaged regions of the brain and attempt to clean up cellular debris (Leonardo and Pennypacker, 2009). Following hypoxia-ischemia, compromise of the BBB (section “Compromise of the Blood Brain Barrier”) allows the entry of macrophages and cytokines into the circulation (Alvarez-Diaz et al., 2007; Leonardo and Pennypacker, 2009). Activated microglia are known to produce inflammatory cytokines interleukin-1β (IL-1β), IL-6 and tumor necrosis factor-α (TNF-α), as well as ROS, and RNS (Kaur and Ling, 2009). Circulating cytokines, which may gain increased entry into the brain through the compromised BBB, may then exacerbate excitotoxicity by again stimulating glutamate release, and free radical and NO production (McLean and Ferriero, 2004).

## Cerebrovascular development in the fetus

Vascularization of the brain begins during embryogenesis, continues into the newborn period, and involves a tightly regulated process of vasculogenesis followed by angiogenesis (Mito et al., 1991; Breier et al., 1992; Ballabh et al., 2004a), when endothelial cells differentiate and proliferate into avascular tissue (Yancopoulos et al., 2000). In the human embryo intra-cerebral blood vessels appear from 7 weeks gestation when the basement membrane of the capillary network forms (Korzhevskii and Otellin, 2000). Newly developed blood vessels are initially large (relatively), irregular in shape, and permeable to hydrophilic substances (Stonestreet et al., 1996), but become thinner and uniformly shaped as the endothelial cells become more tightly interconnected (Engelhardt, 2003).

The germinal matrix, which is abundant with angiogenic vessels (Ballabh et al., 2007; Ballabh, 2010), is the source of neuroblasts from ~10 weeks gestation and glioblasts (oligodendrocyte and astrocyte precursors) in the 3rd trimester (Wigglesworth and Pape, 1978; Volpe, 1989). Heubner's artery is the main vascular channel providing nutrients to this progenitor zone surrounding the lateral ventricle. The germinal matrix is also a terminal area for the medullary artery which extends through to deep white matter (Takashima and Tanaka, 1978b; Wigglesworth and Pape, 1978; Volpe, 1989). By 36 weeks gestation the germinal matrix is nearly involuted when, at this time, cortical regions are undergoing vascular development (Wigglesworth and Pape, 1978; Volpe, 1989). The processes involved in this involution are not well understood, but could be determinants of the increased susceptibility of the germinal matrix to rupture at this time.

The caudate and thalamus develop a distinctive network of fine, circular capillaries in comparison to white matter and subependymal regions, where vessels run perpendicular to the ventricle and have an elliptical shape (Takashima and Tanaka, 1978b). The vascular density of tissue is closely correlated with metabolic demand and hence, cerebral blood flow (Miyawaki et al., 1998), and as gestation proceeds so does vascular density, increasing in the putamen, germinal matrix, and cortex. There are some conflicting findings about the vascularization of the white matter, where Mito et al. (1991) found no change of vascular density during gestation, while others suggest an increase across gestation, (Gould and Howard, 1988; Mito et al., 1991; Miyawaki et al., 1998; Ballabh et al., 2004a), although the change is smaller and the blood vessel density is lower compared to other brain regions.

### Endothelial precursor cells in the fetus

Lineage-committed angioblasts, termed endothelial progenitor cells (EPCs), migrate and congregate into clusters, called blood islands, forming the primary vascular plexus from which a complex microcirculation arises (Risau, 1997). In early embryogenesis, the vascular system develops essentially by vasculogenesis, in which angioblasts differentiate into endothelial cells to form a primitive capillary network, whereas angiogenesis—the sprouting of capillaries from preexisting blood vessels—begins later in embryogenesis and continues throughout fetal life (Risau et al., 1988). EPCs were originally identified as a population of stem cells in human peripheral blood and characterized by the expression of CD34, kinase insert domain receptor (KDR), and CD133 markers (Asahara et al., 1997; Peichev et al., 2000). Subsequently, EPCs have been isolated from bone marrow, fetal liver, and umbilical cord blood. Circulating EPCs are present during the 2nd trimester (Gussin et al., 2002) and seen to increase during gestational age (Sugawara et al., 2005) in pregnant women. Importantly, EPCs are also present in cord blood at various stages of gestation, increasing from low levels at 24–28 weeks to higher levels at 33–36 weeks gestation, equivalent to that found in term infants (Javed et al., 2008). Another study investigating circulating EPCs in preterm infants with bronchopulmonary dysplasia also showed that EPC levels were low at <32 weeks and increased during gestation, and extremely preterm infants with low levels of EPCs had an increased risk of developing bronchopulmonary dysplasia (Borghesi et al., 2009).

### Angiogenesis

Angiogenesis is the process of local proliferation, migration, and remodeling of pre-existing endothelial cells to form new blood vessels, whilst vascular remodeling occurs when an existing vascular network is modified by pruning and vessel enlargement forming interconnecting branches (Yancopoulos et al., 2000; Carmeliet, 2003). Initiation of angiogenesis occurs by the extensive interplay between a variety of cells and numerous growth factors. These include fibroblast growth factor (FGF; both acidic and basic), VEGF, platelet derived growth factor (PDGF), transforming growth factor (TGF) and the angiopoietins (angpt) (Tomanek and Schatteman, 2000). Mitogens, such as VEGF, are activated to enhance endothelial cell migration and proliferation. Endothelial cells migrate along newly deposited extracellular matrix tracts and form vessel sprouts, a process that requires extracellular matrix dissolution facilitated by the presence of proteases such as matrix metalloproteinase (MMP). Finally, tube and basement membrane formation occur triggering further processes which involve the recruitment of pericytes by endothelial cells, and the eventual formation of a “mature” blood vessel (Tomanek and Schatteman, 2000). In the brain, astrocytes induce endothelial cell and pericyte differentiation, but pericytes appear to migrate and cover capillary-like structures faster than astrocytes, which provide the end-feet that eventually invest the outer wall of the blood vessel (Ramsauer et al., 2002). Interestingly, hypoxia is a key regulator of angiogenesis and affects the expression of key angiogenic proteins.

#### Vascular endothelial growth factor and receptors

**Figure 1:**
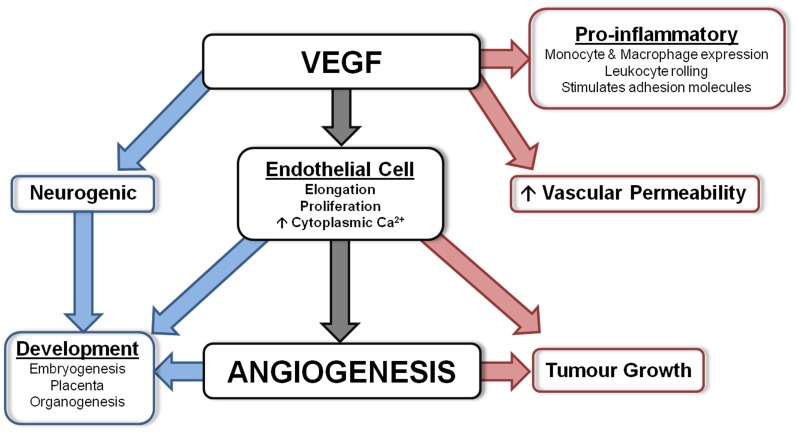
**Diagram summarizing the actions of VEGF.** VEGF is involved in numerous important physiological processes. Angiogenesis is the main function of VEGF, however, it also has protective actions (shown in blue) which include neurogenesis and involvement during development, and detrimental actions (shown in red) including being a pro-inflammatory mediator, vascular permeability factor and involved in tumor growth.

VEGF, first described as vascular permeability factor (VPF) (Keck et al., 1989; Leung et al., 1989), is a key regulator of vasculogenesis and angiogenesis (Breier et al., 1992; Breier and Risau, 1996). During angiogenesis, endothelial cells that express VEGF also produce plasminogen activators and MMPs to initiate extracellular matrix degradation, the first step in angiogenesis as described above. Subsequently, VEGF has an important role in stimulating the proliferation of endothelial cells (Breier et al., 1992). See Figure 1 for a summary.

VEGF has six homologous family members—VEGF-A (previously referred to as VPF, and also referred to as VEGF), placental growth factor (PIGF), VEGF-B, VEGF-C, VEGF-D, and VEGF-E. Three high-affinity tyrosine kinase receptors also exist—VEGFR-1 (Flt-1), VEGFR-2 (fetal liver kinase (Flk-1) in the mouse/KDR in the human) and VEGFR-3 (Flt-4), and are highly expressed on endothelial cells (Breier et al., 1992; Breier, 2000; Carmeliet and Storkebaum, 2002). VEGF-A is the most active, through binding to VEGFR-1 and VEGFR-2 it is involved in blood vessel growth, endothelial cell mitogenesis, vasodilatation (via NO dependent pathways) and vascular permeability (Leung et al., 1989; Breier et al., 1992; Breier, 2000; Dimberg et al., 2010). VEGF-A has seven known isoforms; 121, 145, 148, 165, 183, 189, and 206 (Dvorak et al., 1995; Hoeben et al., 2004). It is the most widely studied of the VEGF family, and in this review article (to keep consistent with the studies referenced), the use of VEGF refers to VEGF-A, unless otherwise stated.

During embryogenesis, the formation of new blood vessels is largely guided by the actions of VEGF through binding with VEGFR-2—responsible for both vascular and neuronal development (Carmeliet, 2003). VEGF and VEGFR-2 activation is also important for promoting neuronal growth, migration, maturation, and survival, and for axonal and dendritic outgrowth (Hermann and Zechariah, 2009; Sentilhes et al., 2010). VEGF is expressed by the embryonic neuroectoderm, the epithelium of the stem cell and progenitor regions of the brain. It has been suggested that during development VEGFR-1 may act as a decoy receptor, but it is a functionally important receptor isoform in the adult, as shown by the effects of hypoxia on the adult rodent cerebral vasculature (Marti and Risau, 1998). VEGF-B and VEGF-C isoforms are present in the brain early in embryonic life (Lagercrantz et al., 1998) and VEGF-C, through binding with VEGFR-3, has recently been shown to be important in the E15.5–18.5 mouse by stimulating growth of non-vascular progenitor cells, such as oligodendrocyte progenitors in the optic nerve (Alonso et al., 2011).

In the developing brain, the temporal and spatial expression of VEGF is correlated closely with vascularization and endothelial cell growth (Breier et al., 1992), and high expression by many cell types (neuroblasts, neuroepithelial, radial glia, astrocytes, pericytes, and endothelial cells) may be a signal coinciding with increasing neuronal metabolism that initiates angiogenesis (Virgintino et al., 2003; Rosenstein and Krum, 2004; Sentilhes et al., 2010). Increased glial VEGF expression occurs as stabilization of the cerebrovasculature is reached, when glial end-feet ensheath blood vessels (Ogunshola et al., 2000).

From 9 weeks gestation, VEGF is strongly expressed in the developing human telencephalon (Virgintino et al., 2003), and from 14 weeks there is strong expression of VEGF and VEGFR-2 by neurons, glia, and blood vessels throughout the brain and cerebellum. During 21–34 weeks gestation VEGF is also strongly expressed in the cortex and white matter (Arai et al., 1998), and by 34 weeks neurons, astrocytes, and endothelial cells weakly express VEGF (Sentilhes et al., 2010). Figure 1 summarizes the numerous suggested physiological actions of VEGF.

#### Angiopoietins

Working in close conjunction with VEGF are the angpt and their ligand receptors Tie (−1 and −2), part of the tyrosine receptor kinases. There are four members of the angpt family, with angpt-1 and angpt-2 the most fully characterized (Yancopoulos et al., 2000; Carmeliet, 2003; Otrock et al., 2007; Hansen et al., 2008). Apart from the known significance of angpt-1 and angpt-2 in tumor growth, in the brain angpt-1 is important for stimulating vessel growth and branching, and is expressed by endothelial cells, astrocytes, and pericytes (Yancopoulos et al., 2000; Carmeliet, 2003; Kim et al., 2008). Angpt-1 knockout mice develop a normal vasculature but are incapable of adequate vascular remodeling. In contrast, angpt-1 over-expression leads to a functional vasculature, but with limited permeability (Suri et al., 1996; Thurston et al., 1999; Yancopoulos et al., 2000), suggesting that angpt-1 may be important for inhibiting vascular permeability.

Angpt-1 also has anti-inflammatory effects, particularly in limiting inflammation-induced vascular leakage and inhibiting neuronal apoptosis (Thurston et al., 1999, 2000; Valable et al., 2003; Hansen et al., 2008). Increased expression of angpt-1 promotes the “tightening” of vessels by increasing expression of tight junction proteins and decreasing permeability and plasma leakage (Thurston et al., 1999, 2000; Valable et al., 2005). Angpt-1 is also implicated in promoting endothelial cell survival, migration, and enhancing the interaction between endothelial cells and neighboring cells that result in the recruitment of pericytes (Suri et al., 1996).

Angpt-2, important in initiating vascular remodeling, has both agonist and antagonist actions to angpt-1 when binding to Tie-2, and co-expression with VEGF may dictate the function of angpt-2, determining whether vessel remodeling, vessel regression occurs (Maisonpierre et al., 1997; Beck et al., 2000; Yancopoulos et al., 2000; Gale et al., 2002). Over-expression of angpt-2 can lead to defective vascular remodeling, as for the angpt-1 knockout mouse (Maisonpierre et al., 1997), and high angpt-2 expression in the absence of VEGF can result in vessel regression, as seen following hypoxia and in tumor growth (Holash et al., 1999; Beck et al., 2000). Expressed mostly by endothelial cells, angpt-2 has been seen to degrade the extracellular matrix and disrupt junctions between endothelial cells in tumor vessels (Gale et al., 2002; Carmeliet, 2003). In the adult mouse brain the presence of angpt-2 and VEGF resulted in increased microvascular density, increased MMP-9 and decreased zona occludin (ZO) expression, leading to increased BBB permeability (Zhu et al., 2005).

### The basement membrane

The basement membrane (also commonly referred to as the basal lamina) exists between endothelial cells and astrocytes (del Zoppo and Mabuchi, 2003). In the brain, it is functionally important in restricting extravasation of protein rich fluids and blood components into the parenchyma (Hamann et al., 1996; Scholler et al., 2007). The basement membrane is composed of collagens and non-collagen glycoproteins (including laminin, and fibrinogen), glycosamineglycans and proteoglycans (heparin sulphate). A direct consequence of the binding of collagen IV and laminin is the tissue support it provides the basement membrane (Charonis et al., 1985; Yurchenco and Schittny, 1990; Hamann et al., 1995; Lukes et al., 1999; Jakobsson et al., 2008). Ment et al. (1991) suggested that basement membrane proteins (laminin and collagen IV) are important in providing structural integrity to blood vessels, particularly in the germinal matrix (subventricular zone) to prevent rupture. Changes in expression are also seen to occur following ischemia (section “Degradation of Basal Lamina”).

Laminin is a non-collagenous constituent of the basement membrane and during development its presence is closely related to the functional and structural maturation of blood vessels (Timpl et al., 1979; Risau and Lemmon, 1988). Laminin is made up of a family of heterotrimeric glycoproteins containing one of 5α, 3β, or 3γ chains, with α-chains being highly bioactive and important in vascular development (Veltkamp et al., 2006; Jakobsson et al., 2008). Normally, laminin expression is confined to the basement membrane, but during development laminin expression is also present in endothelial cells and neuronal and glial precursors, and reactive astrocytes in response to injury (Liesi et al., 1984; Liesi, 1985; Wagner et al., 1997). Angiogenesis is associated with up-regulation of laminin expression and signaling (Risau and Lemmon, 1988; Milner and Campbell, 2002). Regional and time-related variations of expression of the α1, α4, and α5 isoforms in germinal matrix, gray and white matter of both human tissue and rabbit pups may reflect different rates of local angiogenesis (Xu et al., 2008).

Collagen IV is derived from a triple helical molecule, [α1(IV), 2α2(IV)] (Sanes et al., 1990; Yurchenco and Schittny, 1990; Ment et al., 1991) and plays a fundamental role in the maintenance of integrity and function of blood vessels, particularly when there is increased metabolic demand (Poschl et al., 2004). Studies from knockout mice suggest collagen IV is not important until embryonic day 10, while other studies indicate it is important earlier in development, possibly influencing neurite outgrowth (Laurie et al., 1980; Poschl et al., 2004).

Fibronectin is expressed highly during developmental angiogenesis and is essential during embryogenesis (George et al., 1993). Fibronectins are a family of glycoproteins, and important constituents of the basement membrane, promoting endothelial cell adhesion, migration, and organization of the cytoskeleton (Wang and Milner, 2006).

### The blood-brain barrier

The cerebral vascular system has anatomical and physiological barriers that regulate the transfer of compounds into the brain. The major functions of the BBB include protection of the brain from the chemical environment of blood, selective transport of substances into and out of the brain, and metabolism of blood and brain substances (Risau and Wolburg, 1990). The structure of the BBB is composed of capillary endothelial cells, tight junctions, astrocytes, pericytes, and the basement membrane, collectively referred to as the neurovascular unit (Saunders et al., 2008; Abbott et al., 2010). The anatomical barrier lies in the tight junctions that exist between endothelial cells (Brightman and Reese, 1969) that transform into a continuous cellular layer which is specialized to protect the brain. Surrounding the endothelial cells is a basement membrane, pericytes and the astrocytic end-feet that also contribute to barrier function (see below). To complement the cerebrovascular barrier, is a barrier at the epithelial level of the choroid plexus that constitutes the blood-cerebrospinal fluid (CSF) barrier with many similar functions to the BBB. The barrier forming cells at both these barriers also encompass a number of enzymatic systems such as the cytochrome-P450/phase II enzymes, complemented by efflux transporters, which inactivates and reduce the entry of potentially neurotoxic compounds into the brain (Ek et al., 2012). At the same time, nutrients necessary for brain function such as amino acids and glucose are actively transported into the brain.

Barrier properties seem to be established in cerebral vessels as soon as they grow into the brain in early development as tight junctions are formed at this stage (Bauer et al., 1993). However, there has been some debate to the level of maturity and restrictiveness of the barriers in the developing animal. While there have been studies showing less complex tight-junctions in the embryo (Kniesel et al., 1996), others have shown that the tight-junctions are functional early in development (Ek et al., 2003, 2006; Daneman et al., 2010). However, it may be more helpful to view the BBB in relation to the different needs for a developing brain than a simple issue of being less or more restrictive. The developing brain barriers have been shown to be different in many aspects to the adult barriers and include a fetal specific ependymal barrier (Fossan et al., 1985), higher inward transport of both glucose (Cornford and Cornford, 1986) and amino acids (Braun et al., 1980; Tuor et al., 1992) into the developing brain as well as developmental changes in the complement and expression of drug efflux transporters (Ek et al., 2010). However, there are still many undetermined functional aspects of the developing blood-CSF/brain barriers. Some of these differences may very well make the developing brain more vulnerable to various insults, in fact, sheep studies have shown that the BBB becomes less susceptible to hyperosmolaric stress with development (Stonestreet et al., 2006). In rodents the BBB also appears more vulnerable to hypoxia-ischemia at early stages of development (Muramatsu et al., 1997).

#### Tight junctions and adherence junctions

A tight junctional complex exists between the cerebral endothelial cells. They provide a critical structural component that impedes the diffusion of molecules from the blood vessel lumen into the brain parenchyma and are composed of both cytoplasmic proteins (e.g., ZO-1 and 2) and transmembrane proteins such as claudins and occludin (Ballabh et al., 2004b, 2005; Kaur and Ling, 2008). There are also adherence junctions located near endothelial cells holding neighboring cells together. They are composed with cadherin-catenins complex and their associated proteins (Schulze and Firth, 1993).

The claudin family consists of 24 members that bind to each other on adjacent endothelial cells to form a primary seal of tight junctions (Furuse et al., 1999), with claudin-1 and claudin-5 being highly expressed in cerebral capillaries (Furuse et al., 1999). Claudin-5 is expressed as early at 12 weeks gestation in the telencephalon of the human fetus (Virgintino et al., 2004), and only claudin-5 (not claudin-1) is consistently expressed from 16 to 40 weeks gestation in the developing human brain (Ballabh et al., 2005).

Occludin is highly expressed by brain capillary endothelial cells, and their presence is related to increased electrical resistance across the barrier. Occludins appear to be able to alter paracellular permeability with increased expression being indicative of greater barrier properties (Furuse et al., 1993; Hirase et al., 1997). Occludin is expressed as early as 12 weeks gestation in the developing human brain with no change in expression for the duration of pregnancy (Virgintino et al., 2004; Ballabh et al., 2005). These findings show that key tight junctional proteins are present early in human development and imply that functional tight junctions are in place.

There are also a number of other junctional adhesion molecules (JAMs) that are important for tight junction assembly and integrity (Martin-Padura et al., 1998; Bazzoni, 2003). Three JAM proteins exist: JAM-1, JAM-2, and JAM-3, although JAM-2 has not been seen to be expressed in cerebral blood vessels to date (Aurrand-Lions et al., 2001). Ballabh et al. (2005) found JAM-1, but not JAM-2 or JAM-3, was expressed from 16 weeks, with no change of expression across gestation. The most abundant cytoplasmic accessory group of proteins are ZO, important for the structural support of tight junctions (Stevenson et al., 1986).

#### Astrocytes and pericytes

Astrocyte end-feet ensheath blood vessels in the brain, separated from the endothelial cell plasma membrane only by the basement membrane (Risau and Wolburg, 1990; Risau, 1991; Bernstein and Karp, 1995; El-Khoury et al., 2006). This “glial sheath” is important for cerebral vascular structure and function (Bradbury, 1984; Willis et al., 2004). Regions with a discontinuous ensheathment of astrocytes show incomplete or disintegrated basement membrane *in vitro* (Wolff et al., 1974). Absence of the endothelial cell-astrocyte interaction produces areas that are more permeable, such as the circumventricular regions in the hypothalamus and brainstem (Coomber and Stewart, 1984; Goldstein, 1988; Hamm et al., 2004). El-Khoury et al. (2006) investigated astrocyte end-feet coverage in the germinal matrix, white matter and cortex of human fetuses from 16 to 40 weeks gestation using GFAP (a cytoskeleton protein forming the intermediate filament), S-100β (cytosolic calcium binding protein), and aquaporin-4 (AQP4; water channel protein). Remarkably, they found that in the germinal matrix, a region vulnerable to hemorrhage in preterm infants, relatively fewer end-feet and astrocyte processes were labeled with GFAP or S-100β, although this did increase across gestation. The cortex and white matter showed strong perivascular coverage from 16 weeks gestation. However, it should be noted that although astrocyte-endothelial cell contacts are present from very early in brain development, the presence of barrier properties including tight junctional proteins appear to precede these contacts (Daneman et al., 2010), making it more likely that these contacts have regulatory roles at the BBB.

Pericytes are cells that wrap around endothelial cells and provide structural support, stability, and integrity to the vessel wall (Ballabh et al., 2004b; Nakagawa et al., 2007; Kaur and Ling, 2008). Important in vasculogenesis, pericytes are recruited to endothelial cells and are important for both blood vessel and BBB development (Balabanov and Dore-Duffy, 1998). Pericytes are present in the cerebrovasculature from as early at 10 weeks gestation. In comparison to the cortex and white matter, the germinal matrix has fewer pericytes present throughout gestation (Povlishock et al., 1977; Braun et al., 2007), a feature that may be related to the vulnerability of the germinal matrix to hemorrhage in preterm neonates. A distinctive feature of pericytes is their pluripotency, as they exhibit multipotential stem cell activity, phagocytic activity and even express macrophage markers (Balabanov et al., 1996; Dore-Duffy et al., 2006; Bautch, 2011). Findings by Daneman et al. (2010) have clarified the role of the pericytes at the BBB in the developing animal. PDGFR-B null mice have reduced pericyte coverage of cerebral vessels, and this is associated with higher BBB permeability due to increased endothelial vesicular trafficking.

Functionally, pericytes may also be involved in cerebral autoregulation (Hamilton et al., 2010) as they express receptors for, and are modulated by catecholamines, endothelin-1, and vasopressin (van Zwieten et al., 1988; Elfont et al., 1989; Dehouck et al., 1997; Balabanov and Dore-Duffy, 1998; Ballabh et al., 2004b). Pericytes and endothelial cells communicate via gap junctions and their interaction is important for induction of the contractile function that ultimately modulates cerebral blood flow; for detailed reviews see (Hirschi and D'Amore, 1996; Balabanov and Dore-Duffy, 1998; Bergers and Song, 2005; Dore-Duffy, 2008).

## Consequence of hypoxia on cerebral blood vessels

During development any perturbations, such as hypoxia, can significantly alter the expression of key angiogenic genes (Ment et al., 1997; Mu et al., 2003; Kaur et al., 2006a; Keogh et al., 2007) and could thereby result in a dysfunctional vascular system. In the adult brain, severe hypoxia-ischemia such as that seen in stroke may result in breakdown of the endothelial-dependent BBB and physical destruction of the capillary bed, resulting in tissue reactions that include angiogenesis, neovascularization and vascular remodeling (del Zoppo and Mabuchi, 2003), responses that could be seen to be reparative and even neuroprotective. However, the effects of hypoxia on cerebral blood vessels in fetal life has not been widely investigated. Much of what we know is from adult rodent studies and is detailed below.

### Endothelial progenitor cells

In response to hypoxia-sensitive molecular cues, new blood vessel formation, in the adult brain at least, is thought to occur exclusively from angiogenesis. In many adult tissues, hypoxia also induces an inflammatory response within the vessel wall, with subsequent recruitment of circulating EPCs contributing significantly to structural remodeling of the vascular bed. With the discovery of EPCs, it has now been shown that neovascularization after focal cerebral ischemia can also occur via vasculogenesis—the *de novo* process of blood vessel formation by differentiation and migration of EPCs in response to local cues. EPCs can home to local tissue injury and participate in damage repair or wound healing by secreting a number of growth factors that result in neovascularization.

Moreover, systemic administration of cord blood-derived EPCs in adult mice results in significant reduction of infarct volume, decreased neutrophil infiltration, and increased focal blood flow at 48 h after the ischemic insult (Ohta et al., 2006). EPCs have been shown to home to the ischemic core and promote both cerebral neovascularization and neuronal progenitor cell migration and survival (Zhang et al., 2002a), thereby improving cortical expansion after cell transplantation (Taguchi et al., 2004). Furthermore, the pre-existing level of circulating EPCs correlate inversely with cerebral infarction, and positively correlate with regional cerebral blood flow in patients with cerebral ischemia (Ohta et al., 2006), suggesting that EPCs have a functional predictor value for how the cerebral vasculature will be affected by hypoxia-ischemia. Indeed, the level of circulating EPCs and their migratory activity can serve as a marker for the risk of a number of cardiovascular diseases, and as a predictor of vascular function in many diseases (Sen et al., 2011).

Recently, bone marrow-derived EPCs have been shown to be mobilized, recruited and incorporated into ischemic tissue, resulting in neovascularization of ischemic foci in the brain. Furthermore, low circulating EPC level have been found to be predictive of severe neurological impairment by 48 h post-insult, and of the major adverse clinical outcomes on day 90 after acute ischemic stroke, consistent with the finding that increased levels of circulating EPCs after acute ischemic stroke are associated with good functional outcome and reduced infarct growth (Sobrino et al., 2007). Furthermore, hyperoxia impairs EPC signaling and decreases *in vitro* proliferation of EPCs from preterm infants (Fujinaga et al., 2009), decreases vessel density and reduces of circulating EPCs levels in neonatal mice (Balasubramaniam and Del Bigio, 2006), suggesting that oxygen therapy in the newborn period could impair the physiological actions of EPCs.

### Hypoxia-induced angiogenesis and vascular remodeling

In the adult, chronic hypoxia induces angiogenesis, presumably a response aimed at increasing vascularization and oxygen delivery to oxygen-deprived tissue (Shweiki et al., 1993). This process occurs in tumor growth (Kim et al., 1993; Breier and Risau, 1996; Banerjee et al., 1997; Breier, 2000), wound healing (Bates and Jones, 2003; Otrock et al., 2007), diabetic retinopathy (Boulton et al., 1998; Aiello, 2005), and following stroke (Krupinski et al., 1994; Zhang et al., 2000; del Zoppo and Mabuchi, 2003). Neovascularization and vascular remodeling are tightly regulated processes, and in the brain there is a complex interplay of multiple genes; e.g., hypoxia inducible factor-1 (HIF-1α), VEGF and erythropoietin (Epo) expressed by different cell types (Marti et al., 2000). Following stroke, regions of high angiogenesis have also been correlated with increased neuronal survival (Krupinski et al., 1994). Although hypoxia-induced angiogenesis is thought to be a protective in the sense that more oxygen is delivered to hypoxic-ischemic tissue, there are associated effects such as alterations in BBB permeability that are not necessarily beneficial (see below). Several key molecules for regression and re-growth of capillary blood vessels have been identified including VEGF, Placental Growth factor, acidic-FGF, TNF-α, IL-8, and angpts. The consequence of hypoxia-induced angiogenesis in the fetal and neonatal brain is yet to be fully determined.

Angiogenic *remodeling* refers to the process by which this initial network is modified, through both pruning and vessel enlargement in order to form the interconnecting branching patterns characteristic of the mature (stable) vasculature. During this time, vessel walls also “mature” in the sense that endothelial cells become tightly integrated with supporting cells such as smooth muscle cells and pericytes, and with the surrounding matrix. A different process, referred to as angiogenic *sprouting*, involves the formation of new vascular tubes from existing vessels and their penetration into new vascular territory, and this involves degradation of the existing vessel, and the formation of new structures which, being initially incomplete, can be said to be “immature.” The degradation and destabilization of blood vessels, which includes degradation of the basal membrane and extracellular matrix, and also pericyte detachment, can lead to BBB disruption, edema formation, and mechanical rupture, and thus bleeding. The cellular and molecular processes involved vary and depend on the cellular composition of vessels as well as on local environmental factors. Each of the resident vascular cell types (i.e., pericytes, endothelial, smooth muscle) undergo site- and time-dependent changes in proliferation, matrix protein production, expression of growth factors, cytokines, and receptors, and each resident cell type plays a specific role in the overall remodeling response. However, effects of hypoxia on, and vascular responses to fetal hypoxia that cause brain damage are poorly understood.

### Hypoxia inducible factors

In response to hypoxia, HIF-1, a key regulator of oxygen homeostasis is transcriptionally activated to improve oxygen availability (Semenza, 2009b). Increased HIF-1α also contributes to increased expression of angiogenic genes, including VEGF, angpt-2, PDGF-B, and PIGF (Fan et al., 2009; Semenza, 2009b). The robust up-regulation of these genes may act as possible biomarkers in the neonate of a previously undetected hypoxic-ischemic event in the fetus prior to delivery.

Composed of α and β subunits, once in the nucleus, HIF-1α dimerises with HIF-1β and binds to target genes (including Epo and VEGF) at their hypoxia response element (Semenza, 2009b). Three alpha isoforms exist; HIF-1α, HIF-2α, and HIF-3α, although less is known about the actions of the 2α and 3α isoforms compared to HIF-1α. HIF-1α is expressed in many cell types throughout the body whereas, and perhaps importantly for the current discussion, expression of HIF-2α is restricted to vascular endothelial cells (Wiener et al., 1996; Semenza, 2009b; Skuli et al., 2009).

During embryonic and fetal brain development physiological hypoxia stimulates vasculogenesis and angiogenesis, mediated by HIF (Trollmann and Gassmann, 2009). HIF-1α knockout mice show impaired erythropoiesus (among other impairments) and die by embryonic day 8.5 (Iyer et al., 1998; Semenza, 2009a). HIF-2α endothelial deletion shows normal development, but increased vascular permeability, and decreased angpt-2 expression and angiogenesis (SkuLi et al., 2009). It is therefore, suggested that in endothelial cells, HIF-1α may be important for proliferation, metabolism, and survival, whereas HIF-2α could trigger cell migration, adhesion and vascular integrity (Skuli and Simon, 2009).

Following hypoxia in the neonatal brain, HIF-1α predominantly accumulates first in neurons, perhaps because they are most sensitive to fluctuations in oxygen, but sustained high expression of HIF-1α in neurons has been shown to be protective and anti-apoptotic (Fan et al., 2009; Trollmann and Gassmann, 2009). Following neonatal hypoxia-ischemia, mice who had a genetic neuronal knockdown of HIF-1α had worse injury compared to wild type controls (Sheldon et al., 2009). However, inhibition of HIF-1α soon after hypoxia-ischemia has been shown to decrease infarct size, attenuate BBB permeability changes and neuronal death in neonatal rats (Chen et al., 2008), indicating that the actions of HIF-1α are time-dependent.

### Implications of VEGF

**Figure 2:**
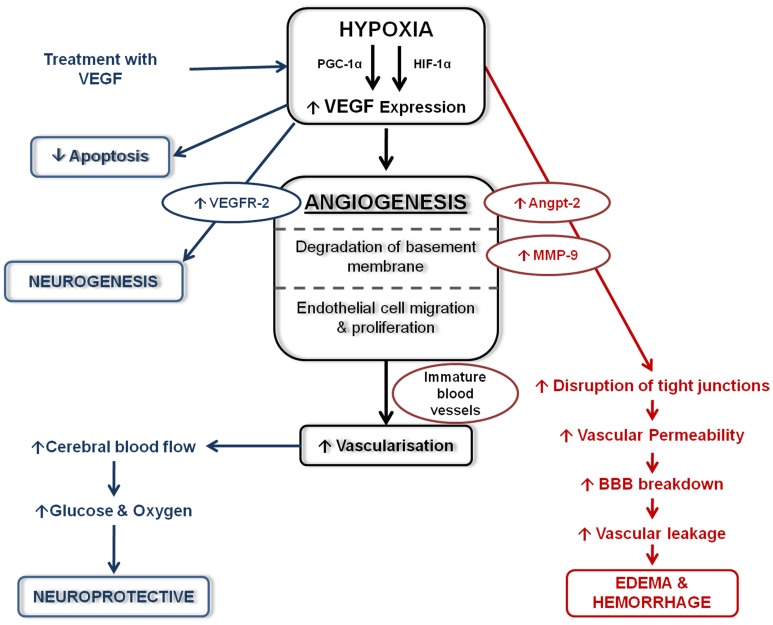
**Schematic diagram of the effects of VEGF following hypoxia.** Following hypoxia, increased VEGF expression stimulated angiogenesis leading to increase vascularization. Increased VEGF expression can have detrimental effects (shown in red) which can lead to edema and haemorrhage formation. Beneficial effects of increased VEGF (shown in blue) help stimulate neurogenesis, decrease apoptosis and are neuroprotective.

The response of VEGF following hypoxia and/or hypoxia-ischemia has been widely studied in the adult (Cobbs et al., 1998; Lennmyr et al., 1998; Plate et al., 1999; Marti et al., 2000; Zhang et al., 2000, 2002b; Sun et al., 2003) and to a lesser extent in the developing brain (Ment et al., 1997; Arai et al., 1998; Mu et al., 2003; Kaur et al., 2006a; Aly et al., 2009). As previously described, a potent stimulator and key transcriptional regulator of VEGF expression is HIF-1α (Forsythe et al., 1996; Marti and Risau, 1998). However, it was recently observed that the up-regulation of VEGF in astrocytes following hypoxia is not entirely HIF-1α-dependent (Schmid-Brunclik et al., 2008), and others have shown that peroxisome-proliferator-activated receptor-gamma coactivator-1alpha (PGC-1α) induces VEGF expression-independently of HIF-1α (Arany et al., 2008). Hypoxia-induced VEGF expression in the brain can have both neuroprotective and neurotoxic actions (Figure 2). This has been recently reviewed by several authors (Carmeliet and Storkebaum, 2002; Skold and Kanje, 2008; Hermann and Zechariah, 2009) and is briefly summarized below.

#### Neurotoxic actions of VEGF

Increased circulating serum VEGF from umbilical cord blood of infants who had experienced birth asphyxia was strongly correlated with hypoxic-ischemic encephalopathy (HIE) and lower Apgar scores (Aly et al., 2009). Elevated circulating VEGF from CSF and placenta samples was also shown to have a strong positive correlation with severity of HIE (Trollmann et al., 2003; Vasiljevic et al., 2011). Arai et al. (1998) also found increased astrocytic and endothelial expression of VEGF in the foci of necrosis in infants who suffered PVL. From postnatal rat studies of either hypobaric (chronic or acute) hypoxia or middle cerebral artery occlusion (MCAO), increased VEGF expression was seen as quickly at 3 h following the insult to being sustained for up to 7 days (Ment et al., 1997; Ogunshola et al., 2000; Mu et al., 2003; Kaur et al., 2006a). Following hypobaric hypoxia in neonatal mice, high VEGF expression is seen together with increased eNOS, iNOS, and nNOS expressions, suggesting a contribution of nitrogen free radicals to both cerebral vasodilation and excitotoxicity (Kaur and Ling, 2009). Astrocytic and neuronal expression of VEGF is high following hypoxia when compared to normoxic controls, high neuronal VEGF expression following hypoxia is also seen in the adult (Marti and Risau, 1998; Ogunshola et al., 2000).

The most profound consequence of increased VEGF expression is the increased vascular permeability, leading to vasogenic edema and the leakage of blood-borne substances into the brain parenchyma (Schoch et al., 2002; Kaur et al., 2006b). In adult models of global hypoxia, focal cerebral ischemia and brain trauma, increased BBB leakage was associated with acute up-regulation of VEGF (Nag et al., 1997; Zhang et al., 2000, 2002b; Schoch et al., 2002; Kaur et al., 2006b), and the increased expression of VEGF in astrocytes is suggested to contribute to this BBB leakage (Schmid-Brunclik et al., 2008). Intravenous administration of VEGF in the newborn mouse has also shown to open the BBB within 2 h (Young et al., 2004). VEGF has a direct action on endothelial cells and pericytes, and pericytes have been implicated in exacerbating disruption of the BBB (Hippenstiel et al., 1998; Yamagishi et al., 1999; Al Ahmad et al., 2009; Thanabalasundaram et al., 2010), possibly because they are amongst the first cells to respond by moving away from the blood vessel wall, and by producing factors such as VEGF which are thought to contribute to vascular instability (Gonul et al., 2002; Al Ahmad et al., 2009; Thanabalasundaram et al., 2010). In brain microvascular endothelial cells, the disruption and disassembly of tight junction proteins occludin and ZO-1 is VEGF mediated (Wang et al., 2001). Fischer et al. (2002) also found that VEGF mediates changes in ZO-1 expression following hypoxia.

#### Neuroprotective actions of VEGF

Hypoxia-induced angiogenesis occurs in an attempt to increase vascularization, to increase cerebral blood flow, and oxygenation in ischemic tissue. VEGF can increase the survival of endothelial cells as well as stimulate and sustain neurogenesis and inhibit apoptosis (Gora-Kupilas and Josko, 2005). Schmid-Brunclik et al. (2008) also found that following hypoxia VEGF contributes to astrocyte proliferation and survival.

Experimental studies from the adult brain suggest that the “early” up-regulation (between 1 h and 3 h post-MCAO) of VEGF could be associated with alterations in BBB permeability and contribute to exacerbating injury. The neuroprotective actions of VEGF including neovascularization and neuronal protection are more likely to occur in the days (after 48 h) following hypoxia (MCAO), when both VEGF and VEGFR-2 have been shown to be up-regulated (Marti et al., 2000). Most interestingly, it is studies that use VEGF treatment that show the best neuroprotective outcome. In both neonatal and adult models following hypoxia, intra-cerebroventricularly injection of VEGF resulted in reduced gross brain injury, subsequently alleviating the decrease in brain weight (i.e., decreased infarct volume), decreased apoptotic cells without increasing BBB permeability. Both studies suggest this is related to the activation of the Akt/ERK pathway (Kaya et al., 2005; Feng et al., 2008). Inhibition of VEGFR-2 has also been shown to decrease endothelial cell proliferation, increase cell death and worsen injury following neonatal stroke in rodents (Shimotake et al., 2010).

Studies from the neonatal rat following hypobaric hypoxia (Kaur et al., 2006a) and MCAO (Mu et al., 2003) have found that VEGF is up-regulated leading to increased vascular permeability as well as possibly being neuroprotective.

### Erythropoietin

Another key hypoxia inducible gene is Epo which, following hypoxia is increasingly important for regulating red blood cell production to increase the capacity of red blood cells to transport and ultimately increase oxygen supply (Marti, 2004; Fan et al., 2009). Hypoxic up-regulation of Epo is regulated by HIF-1α, however, growing evidence suggests HIF-2α may also play a significant role is transcriptionally regulating Epo expression in the brain *in vivo* and in cultured astrocytes *in vitro* (Chavez et al., 2006; Yeo et al., 2008; Fan et al., 2009). Epo is widely expressed in the brain by astrocytes, neurons, microglia, and endothelial cells (Bernaudin et al., 1999; Marti, 2004; Castillo-Melendez et al., 2005). Following cerebral ischemia, endothelial cells are the first to increase Epo expression (Bernaudin et al., 1999), which could implicate Epo as being important for the vasculature following hypoxia. More recently, it has been reported that Epo mediated angiogenesis can occur by stimulating the expression of VEGF and its' receptor on endothelial cells (Iwai et al., 2007; Hermann and Zechariah, 2009). *In vitro*, Epo can modulate angiogenesis by stimulating endothelial cell migration and proliferation (Anagnostou et al., 1990; Yamaji et al., 1996).

Suggested to be neuroprotective following hypoxia, increased Epo expression has also been shown to increase anti-apoptotic gene expression and promote survival in oligodendrocytes, neurons, astrocytes, and microglia (Marti, 2004; Fan et al., 2009). Treatment with recombinant human Epo following focal hypoxia-ischemia in neonatal rats results in enhanced revascularization, neurogenesis, endothelial cell and neuronal survival and increased Glut-1, Tie-1, and angpt-2 expression which resulted in enhanced neurovascular unit repair (Iwai et al., 2007; Keogh et al., 2007). Epo treatment following neonatal stroke in rats has also shown significant neuroprotection (Chang et al., 2005; Gonzalez et al., 2007).

### Angiopoietins

There is a close interaction between VEGF and angpt (−1 and −2) expression following hypoxia. Down-regulation of angpt-1, important for blood vessel stabilization, occurs in response to hypoxia, and this correlates with increased BBB permeability and vascular leakage (Zhang et al., 2002b; Valable et al., 2005). Whether vascular remodeling or regression occurs following cerebral ischemia in the adult (rat) is dependent upon the presence of VEGF. Increased angpt-2 and VEGF expression by blood vessels promotes vascular growth; however, vascular regression occurs in the absence of VEGF (Beck et al., 2000). Up-regulation of Tie-1 and Tie-2, the angpt receptors, persists for up to 2 weeks following MCAO and this correlates closely with increased angpt-1 and angpt-2 expression resulting in neovascularization (Lin et al., 2000, 2001). However, increased angpt-2 expression can also persist with no change in angpt-1 or Tie-2 expression following MCAO, or during chronic hypobaric hypoxia in the adult rat (Beck et al., 2000; Pichiule and LaManna, 2002).

### Degradation of basal lamina

**Figure 3:**
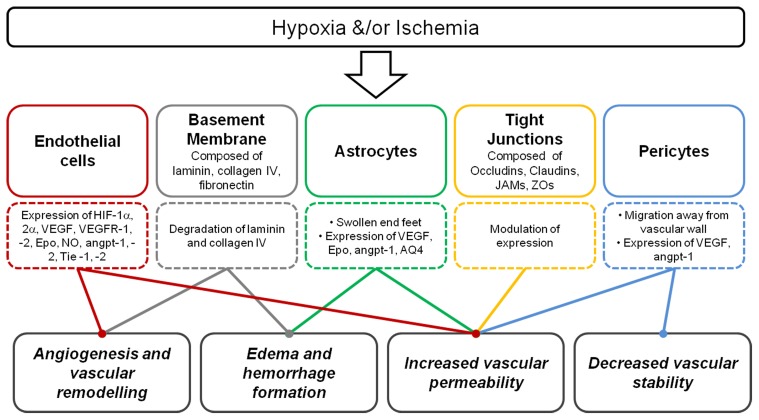
**Summary of the consequence of hypoxia and/or ischemia on the neurovascular unit.** Following hypoxia, endothelial cells express a range of key hypoxic and angiogenic factors, basement membrane degradation occurs, astrocyte end-feet become swollen, modulation of tight junction expression occur and pericytes may migrate away. These responses contribute to angiogenesis, vascular remodeling, edema, and hemorrhage formation, increased vascular permeability, and decreased vascular stability.

Degradation of laminin is consistent with disruptions of the BBB (Figure 3) and can contribute to edema formation and hemorrhage (del Zoppo and Mabuchi, 2003; Veltkamp et al., 2006). Degradation of the basal lamina following hypoxia is due to plaminogen-plasmin system and the MMP-2 and 9. MMPs belong to a family of proteins that control tissue degradation (Hamann et al., 1995; Burk et al., 2008). Following hypoxia-ischemia, MMP-9 is up-regulated, and laminin being a substrate, is thought to result in the laminin degradation that may then follow (Zalewska et al., 2002). Following ischemia, decreased collagen IV is associated with increased infarct volume and changes in BBB permeability (Hamann et al., 1995, 2002; Scholler et al., 2007). However, another basement membrane constituent fibronectin, and its' α5β1 integrand receptor, are also up-regulated in endothelial cells following hypobaric hypoxia in the adult, possibly highlighting an angiogenic role for this glycoprotein (Milner et al., 2008).

### Compromise of the blood brain barrier

Studies in rodent models of neonatal hypoxia-ischemia have indicated that the BBB is compromised as a result of such insults (Muramatsu et al., 1997; Svedin et al., 2007; Ferrari et al., 2010b; Tu et al., 2011; Yang et al., 2012). In addition, CSF samples from HIE infants have been shown to have albumin CSF/plasma concentration ratios that are ~5 times higher than normal (Kumar et al., 2008), suggesting a dysfunctional BBB in the HIE infants. The animal studies have mostly relied on the extravasation of plasma proteins into brain parenchyma to examine BBB breakdown making the exact timing and magnitude of breakdown after the insult difficult to ascertain. Ferrari et al. (2010b) who used sodium fluorescein uptake into the brain to assess barrier permeability, reported that after hypoxic-ischemic insult in P7 rats there was an increase in BBB permeability at 1 and 7 days after the insult, but it was restored to normal at 21 days. All these studies do suggest that hypoxia-ischemia related injury to the developing brain is associated with compromise to the barrier, however, what role this has in determining the pathological outcomes is still unclear.

Modification of tight junction proteins may be a cause of changes to BBB permeability following hypoxia-ischemia (Figure 3). Malaeb et al. (2007) determined the level of tight junctional proteins after cerebral ischemia in late gestational sheep. Cortical samples showed that these proteins were differentially regulated after the insult with higher claudin-5 levels, but lower ZO-1/2 levels. Adult studies of cerebral ischemia-reperfusion have also shown that this is associated with changes to the levels of tight junction proteins in cerebral vessels (Witt et al., 2003). Studies in rats by Muramatsu et al. (1997) showed that the BBB was more vulnerable to a hypoxic-ischemic insult in younger (P7 day) compared to older (P21 day) rat pups, and as mentioned above, this susceptibility may be related to several developmental aspects of the formation of the neurovascular unit. The severity of oxygen deprivation contributes to the rate of loss of BBB function during acute hypoxic exposure (Al Ahmad et al., 2009). However, in a model of hypoxia in the newborn piglet BBB integrity, measured as permeability to small ions, was maintained (Stonestreet et al., 1992).

Figure 3 summarizes the consequence of hypoxia on the components of the neurovascular unit. As mentioned previously, increased VEGF expression following hypoxia-ischemia results in compromise of BBB permeability. Synthesis and release of soluble guanylate cyclase, NO, tissue type plasminogen activator, prostaglandins (PG) and increased calcium influx all contribute to changes in vascular permeability (Bates and Curry, 1997; Murohara et al., 1998; Mayhan, 1999; Yang et al., 2009).

Pericytes are some of the first cells to respond to hypoxia, within the hours following hypoxia in the adult, some pericytes also migrate away from vessels (Gonul et al., 2002) resulting in decreased vascular support. *In vitro* and *in vivo* studies have shown that pericytes can express VEGF and exacerbate BBB disruption following acute hypoxia (Yamagishi et al., 1999; Al Ahmad et al., 2009, 2011). Pericytes can also express angpt-1, but whether pericyte migration occurs in the fetal brain following hypoxia, in regions of BBB compromise could reveal the basis of vascular fragility (leading to an increased propensity to rupture).

#### Edema formation

Following hypoxia, BBB leakage triggers the activation of astrocytes, corresponding with an increase in AQP4, an astroglial water channel which facilitates water movement in and out of the brain, a response that is likely to be involved in the formation of edema (Kaur et al., 2006b; Ferrari et al., 2010a,b). Two forms of edema exist; cytotoxic and vasogenic edema. Cytotoxic edema is swelling of cellular elements and is suggested to occur in severe cardiovascular and hypoxic collapse seen following pneumothorax (Temesvari et al., 1984; Klatzo, 1987). Vasogenic edema is associated with increased vascular permeability, retention of water and entry of serum proteins into the brain parenchyma (Klatzo, 1987). Disruptions in BBB permeability, elevation in mean arterial blood pressure and subsequent accumulation of water content into the brain parenchyma all contribute to the formation of vasogenic edema (Kuroiwa et al., 1985; Klatzo, 1987).

Utilizing MRI, clinical studies have found that following severe perinatal asphyxia more than 80% of term neonates develop brain edema (Boichot et al., 2006), which has also been shown to be strongly associated with poor neurological outcome (Chang et al., 2007). From rodent studies, neonatal hypoxia-ischemia resulted in a biphasic edema formation occurring initially as early as 2 h and persisting after 24 h. This was seen to correspond with an “early” neuronal injury and “late” glial damage (Nedelcu et al., 1999).

## Current clinical therapies and the influence on cerebral blood vessels

As well as understanding the response of cerebral blood vessels following hypoxia-ischemia, it is importantly to briefly detail the implications of the current clinical therapies given for preterm birth and possibly reduced injury following hypoxia-ischemia.

### Glucocorticoids

Prenatal glucocorticoids are routinely administered to women who are at risk of preterm birth, in an attempt to hasten fetal lung maturation, especially with respect to surfactant production. They have been shown to reduce respiratory distress syndrome and intraventricular hemorrhage (Liggins and Howie, 1972; Shankaran et al., 1995). The possible mechanisms that may contribute to this protection have been elegantly investigated by Vinukonda et al. (2010). They found that glucocorticoid treatment resulted in decreased endothelial proliferation and vascular density but increased pericyte coverage in the germinal matrix of human preterm infants. *In vivo* studies in neonatal rats have shown that dexamethasone treatment prior to hypoxia-ischemia increased VEGF protein, reducing brain injury, decreased caspase-3 activity and DNA fragments (Feng et al., 2011). Interestingly, maternal treatment with glucocorticoids such as dexamethasone has shown to also up-regulate key tight junctional proteins and decrease BBB permeability in the ovine fetus (Stonestreet et al., 1999; Sadowska et al., 2010), consistent with *in vitro* studies that also show up-regulation of angpt-1 and down-regulation of VEGF in astrocytes and pericytes following dexamethasone treatment (Kim et al., 2008). However, dexamethasone treatment had no effect on expression of angpt-1, -2 or VEGF expression by endothelial cells (Kim et al., 2008). The implications of these studies are that glucocorticoids, routinely used to mature the preterm lung, could have direct beneficial effects on the preterm cerebral circulation. However, it must be considered also that the greater ease with which preterm neonates are resuscitated and ventilated may mean that the mechanical and cardiorespiratory disturbances are lessened, so that wide fluctuations of cerebrovascular perfusion are somewhat avoided.

### Hypothermia

Brain and/or body cooling is becoming a routine practice for infants that have suffered birth asphyxia. Hypothermia decreases cerebral metabolism, and thereby delays cell death. One study has shown that following permanent MCAO in the adult rodent, mild hypothermia resulted in decreased infarct size and enhanced angiogenesis (Xie et al., 2007). It has been suggested that hypothermia may therefore, play a beneficial role with regards to angiogenesis in the sub-acute (hours to days) and chronic (weeks to months) stages following hypoxia-ischemia (Yenari and Han, 2012). There are also studies indicating that hypothermia restores BBB function after hypoxia-ischemia, but this seems only to have been studied after ischemia in the adult. Hypothermia can reduce the activation of MMPs (Truettner et al., 2005) which can degrade tight junctional proteins and it can also delay pericyte migration away from the endothelial cell (Duz et al., 2007).

## Summary

This review summarizes current knowledge regarding the response of the vasculature and the BBB in the fetal and newborn brain to hypoxia and/or ischemia. Although the studies of the developing brain are limited in comparison to the adult brain, the findings indicate that hypoxia-ischemia has significant effects on the immature cerebral vasculature, perhaps explaining some of the regional vulnerability of the perinatal brain to injury. The key mediators of injury mentioned in this review also reveal great therapeutic potential, not only for early biomarkers of brain injury, but also for designing clinical treatments that might be used both prior to or following hypoxia to protect the immature brain from permanent damage.

### Conflict of interest statement

The authors declare that the research was conducted in the absence of any commercial or financial relationships that could be construed as a potential conflict of interest.
